# Pseudomyogenic hemangioendothelioma of bone treated with denosumab: a case report

**DOI:** 10.1186/s12885-019-6072-8

**Published:** 2019-09-03

**Authors:** Shinya Otani, Robert Nakayama, Tetsuya Sekita, Toru Hirozane, Naofumi Asano, Kazumasa Nishimoto, Aya Sasaki, Hajime Okita, Hideo Morioka, Masaya Nakamura, Morio Matsumoto

**Affiliations:** 1 Department of Orthopaedic Surgery, Keio University School of Medicine, 35 Shinanomachi, Shinjyuku, Tokyo, 160-8582 Japan; 2 Department of Diagnostic Pathology, Keio University School of Medicine, Tokyo, Japan; 3 Department of Pathology, Keio University School of Medicine, Tokyo, Japan

**Keywords:** Pseudomyogenic hemangioendothelioma, Denosumab, Osteoclast-like giant cells, FOSB

## Abstract

**Background:**

Pseudomyogenic hemangioendothelioma (PMHE) is a rare endothelial neoplasm that involves the bones in only 14% of all cases. The optimal treatment strategy has not been established. We herein report a case of primary PMHE in which denosumab treatment showed activity in both imaging studies and the clinical outcome.

**Case presentation:**

A 20-year-old woman presented with worsening pain in her left ankle. Imaging studies showed multifocal fluorodeoxyglucose (FDG)-avid [maximum standardized uptake value (SUVmax), 15.95] osteolytic lesions in the bones of her left lower extremity. While waiting for the definitive pathologic diagnosis of PMHE, denosumab, a human immunoglobulin G2 monoclonal antibody against RANKL, was initiated to treat progressive bone absorption after curettage of one of the lesions. Denosumab induced osteosclerosis around the lesions and pain relief and was discontinued 4 years after its initiation. Although all of the multifocal lesions remained, they all became less FDG-avid (SUVmax, 2.6), and the patient developed no signs of new lesions or distant metastasis.

**Conclusion:**

Denosumab plays a certain role in prevention of bone destruction by PMHE through suppression of osteoclast-like giant cells and would be an excellent treatment for bone absorption by PMHE of bone.

## Background

Pseudomyogenic hemangioendothelioma (PMHE) is a rarely metastasizing endothelial neoplasm that most commonly occurs in young adults and often presents as multiple discontiguous nodules in different planes of a limb. This extremely rare vascular tumor was newly classified in the World Health Organization (WHO) classification of Tumors of Soft Tissue and Bone in 2013^1)2)^. The histological diagnosis of PMHE is often challenging, the optimal treatment has not been established, and the long-term prognosis is unclear. We herein report a case of 20-year-old woman with multiple intraosseous PMHE lesions in her left lower extremity that showed rapidly progressive osteolytic change. Although the multiple lesions did not disappear, denosumab administration led to pain relief, attenuation of the tumor viability, and formation of clear bone sclerosis around the lesions.

## Case presentation

**Fig. 1:**
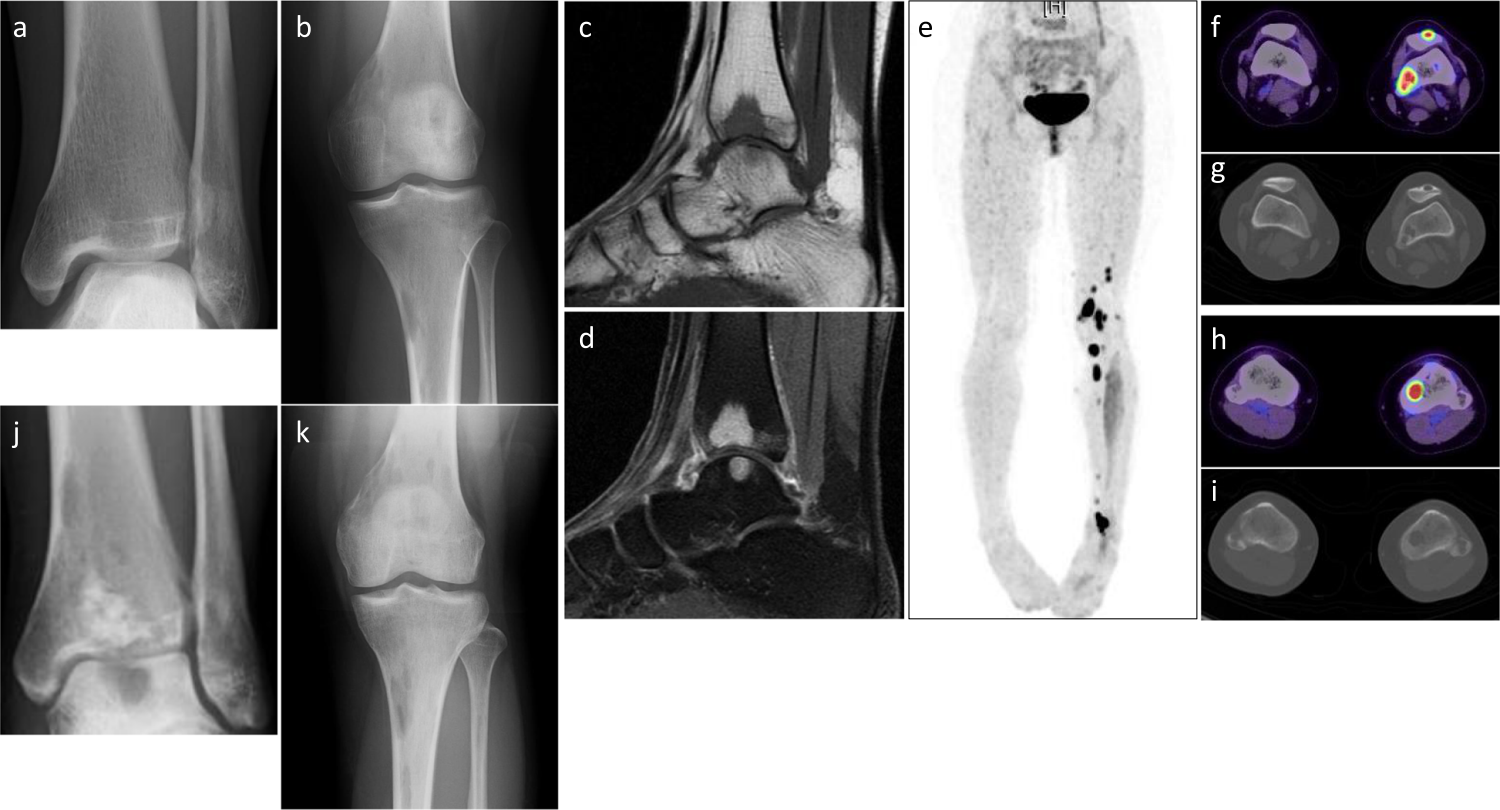
Imaging findings at presentation. **a** and **b** Plain X-ray findings. Multifocal ill-circumscribed lytic lesions were seen in the left femur, patella, tibia, and talus. **c** and **d** Sagittal magnetic resonance imaging findings of the left ankle. The multifocal lesions showed **c** homogeneous low intensity on T1-weighted images and **d** homogeneous enhancement after gadolinium administration. **e-i** Fluorodeoxyglucose (FDG)-positron emission tomography/computed tomography showed multifocal FDG-avid lesions in the bones of the left lower extremity with a maximum standardized uptake value of 15.95. No lesions were found in the soft tissue. **j** and **k** Rapid progression after curettage and artificial bone grafting of the lesion at the distal end of the left tibia was observed. Each ill-circumscribed lytic lesion in the left extremity increased in size over 1 month

**Fig. 2:**
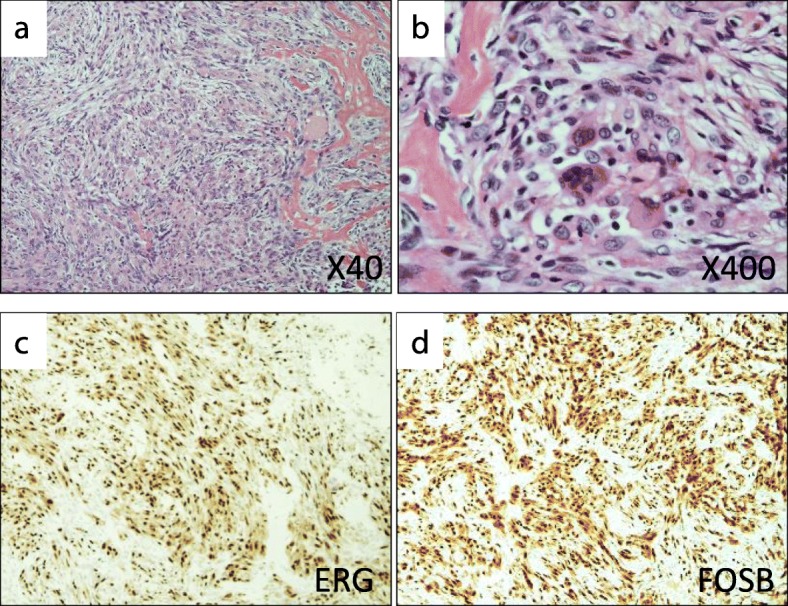
Pathological findings. **a** and **b** Hematoxylin–eosin staining showed proliferation of spindle and epithelioid cells with eosinophilic cytoplasm, which was compatible with the diagnosis of pseudomyogenic hemangioendothelioma. **c** and **d** Immunohistochemical studies showed that the tumor cells were positive for ERG and FOSB

**Fig. 3:**
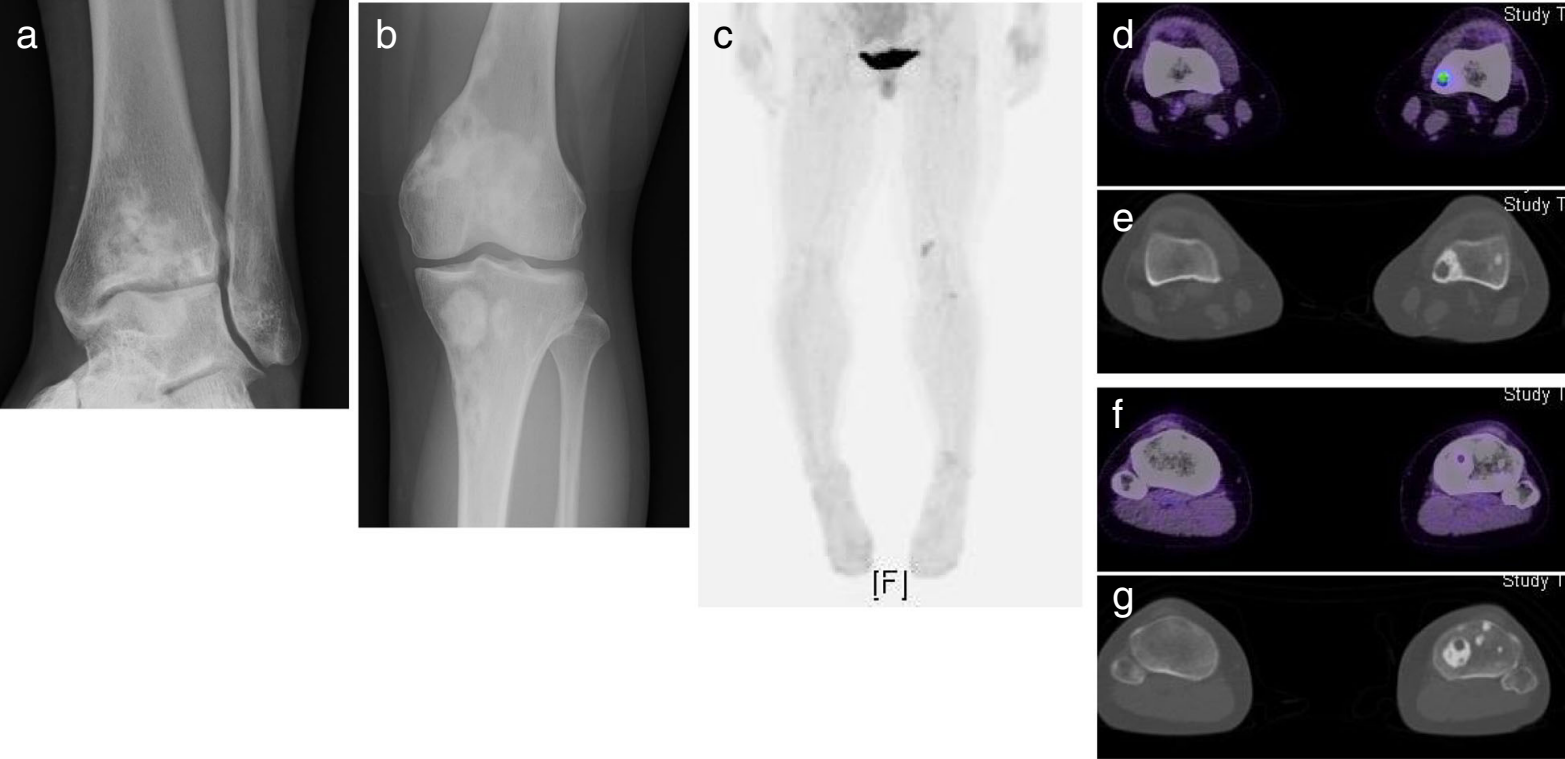
Imaging findings 4 years following denosumab treatment. **a** and **b** Plain radiographs showed shrinkage of the lesions with striking marginal sclerosis. **c-g** Fluorodeoxyglucose (FDG)-positron emission tomography/computed tomography showed that each lesion had become less FDG-avid with a maximum standardized uptake value of 2.6 and was accompanied by remarkable sclerotic bone

A 20-year-old woman with no medical history was referred to our hospital for evaluation and treatment of a 3-month history of worsening pain in the left ankle. Physical examination revealed no remarkable findings except tenderness on the anterior aspect of her left ankle. All laboratory data were within the reference ranges. Radiographs showed multiple discontiguous osteolytic lesions in the bones of the left lower extremity (femur, patella, tibia, and talus) (Fig. 1a, b). The spotty lytic lesions were ill-circumscribed and lacked marginal sclerosis. Some were accompanied by thinning and ballooning of the bone cortexes (Fig. 1a, b). The lesion at the distal end of the tibia extended beyond the epiphyseal plate and eroded the subchondral bone; this lesion was considered to be the cause of her ankle pain. On ankle magnetic resonance imaging, the spotty lesions showed isointensity to the muscles on T1-weighted images (Fig. 1c), mild hyperintensity on T2-weighted images, and homogeneous enhancement by gadolinium administration (Fig. 1d). On fluorodeoxyglucose positron emission tomography (FDG-PET), all of the intraosseous spotty lesions in the left lower extremity were FDG-avid with a maximum standardized uptake value (SUV) of 15.95; no such lesions were present in the soft tissues (Fig. 1e–i). Based on the findings from the imaging studies, the differential diagnoses of the multiple bone lesions included bone metastasis, hematological malignancies such as malignant lymphoma and multiple myeloma, and vascular tumors. Curettage of the lesion in the distal tibia and artificial bone grafting were performed for pain relief and histological diagnosis. While waiting for the definitive diagnosis, progressive bone absorption at all of the lesions and worsening lower limb pain occurred over 1 month (Fig. 1j, k). The preliminary pathology report suggested a non-hematological neoplasm composed of spindle cells and osteoclast-like giant cells, which led to a clinical diagnosis of bone metastases from a solid cancer despite the fact that no lesion suspicious of primary cancer was present. Based on the clinical diagnosis of bone metastasis, monthly administration of denosumab (120 mg) was initiated. The final pathological report demonstrated proliferation of spindle cells and epithelioid cells with eosinophilic cytoplasm. The tumor cells were immunohistochemically positive for keratin (AE1/AE3), CD31, ERG, and FOSB (Fig. 2a–d), which led to the diagnosis of PMHE. Because her limb pain gradually improved after denosumab administration, the monthly denosumab treatment was continued. After 1 year of denosumab treatment, the drug was administered at longer intervals of up to 6 months. The drug was discontinued 4 years after its initiation. Although the spotty lesions remained, a plain radiograph showed no increase in the size of the lesions or the surrounding marked marginal scleroses (Fig. 3a, b). On FDG-PET, the lesions showed clearly decreased SUVs (Fig. 3c–g). Because denosumab treatment led to dramatic symptom improvement, the patient did not agree to undergo a second-look biopsy or curettage of the remaining lesions at the time of denosumab discontinuation. The patient developed no signs of new lesions or distant metastasis and agreed to continue undergoing active surveillance.

## Discussion and conclusions

PMHE is an extremely rare vascular tumor that was first reported by Mirra et al. [1] as a fibroma-like variant of epithelioid sarcoma in 1992. It was first defined by Hornick and Fletcher [2] in 2011 and newly classified in the 4th edition of the WHO classification of soft tissue tumors in 2013 [3]. Hornick and Fletcher [2] reported the largest case series involving 205 total lesions in 50 patients, which showed a striking male predominance (4.6:1.0) and a relatively young age at onset (mean, 31 years; 82% of patients were ≤ 40 years old). Nearly half of the tumors arose in the lower extremities (54%), and two-thirds (66%) were multifocal lesions (range, 2–15 lesions). The lesions involved multiple tissue planes in 64% of cases, including the dermis, subcutis, muscle, and bone. Only 14% of the tumors were in the bones, and the tumor involved the bone alone in only one case [2]. Inyang et al. [4] reported a case series of 10 patients with primary PMHE of bone and found patient demographics similar to those described by Hornick and Fletcher [2]. Plain radiographs showed multiple discontinuous osteolytic and circumscribed lesions, and pathological findings showed that 60% of the tumors had osteoclast-like giant cells [4]. Although the lesions were in one lower extremity, where PMHE commonly occurs, the present case was exceptional in terms of the sex of the patient (female) and the tissue plane involved (bone alone). Twenty cases of primary bone PMHE, including the present case, have been reported in the English-language literature (Table 1) [2, 4–12]. Most of the tumors were initially thought to be multiple bone metastases or multiple myeloma and were subsequently diagnosed as PMHE. Solitary lesions were seen in only three cases (15.0%). Multiple lesions in a lower extremity were seen in half of the cases (10/20). No definitive evidence distinguished multiple bone metastases of PMHE from the multifocal discontiguous primary lesions that are often seen in vascular tumors.

**Table 1:** Cases of primary bone pseudomyogenic hemangioendothelioma

Case	Author	Year	Age	Sex	Location	Laterality	Comments
1	Hornick	2011	35	Male	Finger	Solitary	
2	Sheng	2012	10	Female	Lower extremety, multiple	One-sided	
3	McGinity	2013	25	Male	T4 spine	Solitary	
4	Bryanton	2015	59	Male	Multiple	Both	
5	Righi	2015	66	Female	Lower extremety, multiple	One-sided	Curettage
6	Shah	2015	86	Male	Lower extremety, multiple	One-sided	Pulmonary mets
7	Joseph	2015	45	Male	Illum	Solitary	
8	Inyang-1	2016	59	Male	Multiple	Both	Complete metabolic resolution
9	Inyang-2	2016	19	Male	Lower extremety, multiple	One-sided	
10	Inyang-3	2016	47	Male	Lower extremety, multiple	One-sided	
11	Inyang-4	2016	14	Male	Upper extremety, multiple	One-sided	
12	Inyang-5	2016	74	Male	Trunk, multiple	One-sided	Spinal metastasis
13	Inyang-6	2016	20	Male	Femur, multiple	One-sided	
14	Inyang-7	2016	66	Male	Trunk, multiple	Both	
15	Inyang-8	2016	12	Male	Lower extremety, multiple	One-sided	
16	Inyang-9	2016	26	Male	Multiple	Both	Brain metastasis
17	Inyang-10	2016	5	Female	Hip and pelvis, multiple	One-sided	
18	Krebs	2017	33	Male	Lower extremety, multiple	One-sided	
19	Ozeki	2017	15	Male	Lower extremety, multiple	One-sided	Treated with everolimus. Spinal metastases
20	Present		20	Female	Lower extremety, multiple	One-sided	

The extreme rarity of this vascular tumor and its less characteristic morphological features make the histological diagnosis of PMHE enormously challenging. PMHE is known to be immunohistochemically positive for keratin AE1/AE3 and the endothelial transcription factor ERG. The balanced translocation t(7;19)(q22;q13), resulting in a *SERPINE1-FOSB* fusion, was recently identified in all cases of PMHE [13, 14]. This consistent genetic alteration is considered to be the cause as well as a useful diagnostic marker of PMHE, and the positivity of FOSB by immunohistochemistry is an excellent surrogate marker for the presence of the genetic alteration [15, 16]. In the present case, the immunohistochemical positivity for FOSB in addition to CD31 and ERG finally led to the diagnosis of PMHE.

The optimal treatment for PMHE has not been established to date, and the long-term oncological outcome remains unclear. A wide variety of treatments have been conducted, including surgery, chemotherapy, and the combination of both. In terms of surgery, Hornick and Fletcher [2] reported that all tumors had infiltrative margins and that 58% of patients developed local recurrence or additional nodules in the same region after local excision. Despite the generally indolent nature of PMHE, amputations were required for local control in some cases [2, 4], and systemic progression was seen in other cases. Conversely, two cases of spontaneous regression have been reported [4, 7]. In terms of chemotherapy, the efficacy of gemcitabine and docetaxel and everolimus [10, 12] has been reported.

Denosumab is a human immunoglobulin G2 monoclonal antibody specific to receptor activator of nuclear factor kappa-B ligand (RANKL) and has been used for the treatment of osteoporosis, bone metastases, and giant cell tumors of bone [17–19]. In this case, all lesions showed simultaneous rapid progression of bone destruction that resulted in exacerbation of the limb pain after curettage. Denosumab was administered under the clinical diagnosis of bone metastasis to prevent further bone destruction. Given the indolent nature of the disease after diagnosis and the fact that most patients in the literature presented with pain associated with bone destruction, it is likely that each PMHE tumor of bone is induced to progress at some point by unknown triggers in its natural course. Reactive osteoclast-like giant cells have been seen in more than 60% cases [4], which are thought to be activated by FOSB-positive tumor cells by the unknown mechanism and considered responsible for the lytic destruction of the bone. Although denosumab did not show an essential anti-tumor effect because all of the lesions remained, we believe that denosumab played a certain role in suppression of the bone absorption by osteoclast-like giant cells and subsequent pain relief. The formation of highly characteristic osteosclerosis around the lesions could be the corroborating evidence. The precise mechanism of denosumab’s activity in PMHE of bone was not clear since the tumor was not evaluated histologically after denosumab treatment.

Despite strikingly decreased SUVs, FDG-PET still showed multiple discontiguous nodules 4 years after initiation of denosumab treatment. The stable oncologic course of the case could be attributed to the indolent nature of the tumor. Deciding the optimal duration of denosumab treatment in such a young female patient with possibly indolent disease was enormously difficult. Denosumab was gradually withdrawn considering the substantial risk of osteonecrosis of the jaw due to the years-long denosumab administration, and also considering the teratogenic potency of the drug for future pregnancy. The patient developed no signs of new lesions, distant metastasis or progression of the bone absorption even after the termination of denosumab. Close long-term follow-up is still necessary, and denosumab treatment will be considered if the bone absorption recur.

## Data Availability

All relevant data are provided in the manuscript.

## Abbreviations

FDG: Fluorodeoxyglucose
PET: Positron emission tomography
PMHE: Pseudomyogenic hemangioendothelioma
RANKL: Receptor activator of nuclear factor kappa-Β ligand
SUVmax: Maximum standardized uptake value
WHO: World Health Organization
